# Increased Cytoplasmic Localization of p27^kip1^ and Its Modulation of RhoA Activity during Progression of Chronic Myeloid Leukemia

**DOI:** 10.1371/journal.pone.0076527

**Published:** 2013-10-01

**Authors:** Anita Roy, Lakshmishri Lahiry, Debasis Banerjee, Malay Ghosh, Subrata Banerjee

**Affiliations:** 1 Structural Genomics Division, Saha Institute of Nuclear Physics, Kolkata, West Bengal, India; 2 Department of Haematology, Ramkrishna Mission Seva Pratisthan, Kolkata, West Bengal, India; 3 Department of Haematology, N R S Medical College and Hospital, Kolkata, West Bengal, India; B.C. Cancer Agency, Canada

## Abstract

The role of p27^kip1^ in Chronic Myeloid Leukemia (CML) has been well studied in relation to its function as a cell cycle inhibitor. However, its cytoplasmic function especially in CML remains to be seen. We studied the localization of p27^kip1^ and its function during the progression of CML from chronic to blast phase. Our investigations revealed an increased localization of p27^kip1^ in the cytoplasm of CD34^+^ cells in the blast phase compared to chronic phase. Cytoplasmic p27^kip1^ was found to modulate RhoA activity in CD34^+^ stem and progenitor cells. Further, RhoA activity was shown to be dependent on cytoplasmic p27^kip1^ which in turn was dependent on p210^Bcr-Abl^ kinase activity. Interestingly, RhoA activity was observed to affect cell survival in the presence of imatinib through the SAPK/JNK pathway. Accordingly, inhibition of SAPK/JNK pathway using SP600125 increased apoptosis of K562 cells in presence of imatinib. Our results, for the first time, thus reveal a crucial link between cytoplasmic p27^kip1^, RhoA activity and SAPK/JNK signalling. To this effect we observed a correlation between increased cytoplasmic p27^kip1^, increased RhoA protein levels, decreased RhoA-GTP levels and increased SAPK/JNK phosphorylation in blast phase CD34^+^ cells compared to chronic phase CD34^+^ cells.

## Introduction

Chronic Myeloid leukemia (CML) is a clonal myeloproliferative disorder characterized by the presence of p210^Bcr-Abl^ fusion protein with a constitutively active tyrosine kinase activity [1]. The disease progresses from an initial chronic phase to accelerated phase and finally to an advanced blast phase where greater than 20% myeloid and lymphoid lineage blast cells are found in the peripheral blood. Blast phase CML patients are known to harbor therapy-refractory and differentiation-arrested cells [2]. Resistance to standard treatment in blast phase CML has been attributed to increased genomic instability, increased frequency of point mutations within the kinase domain of p210^Bcr-Abl^ and acquisition of new tumor suppressor and oncogenic mutations [3]. Blast crisis CML thus remains a sordid reminder of the limitations of therapy and therefore a better understanding of the molecular events leading to blast phase CML is required for building a robust treatment regime.

Previous studies have conclusively demonstrated that p210^Bcr-Abl^ is required for uncontrolled proliferation [4,5] and decreased apoptosis [6,7], all characteristics of CML cells. A large body of research shows that cell cycle is tightly regulated by cyclin-dependent kinases and their regulatory inhibitors (CDKIs). A prominent CDKI involved in the regulation of G1-S phase transition is p27^kip1^. It interacts with the Cdk2-cyclinE and Cdk2-cyclinA complexes and thereby regulates the activity of these kinases [8,9]. p210^Bcr-Abl^ has been shown to promote cell cycle progression by down regulating the expression of p27^kip1^ [10]. Furthermore, p210^Bcr-Abl^ also induces the expression of Skp2 and thus promotes the degradation of p27^kip1^ [11,12]. Another mode of regulation involves p210^Bcr-Abl^ induced mislocalization of p27^kip1^. All these processes enable p210^Bcr-Abl^ to control cell cycle progression [13,14]. Thus, p27^kip1^ has emerged as a possible player in CML management [15].

Previous studies have indicated the role of p27^kip1^ outside the nucleus, i.e. in the cytoplasm. The cytoplasmic localized p27^kip1^ has been associated with actin cytoskeleton remodeling [16]. Cytoplasmic mislocalization of p27^kip1^ has also been associated with aggressive metastatic forms of cancer [17,18]. p27^kip1^ is thought to mediate these effects through its interaction with RhoA [19,20]. A plausible p27^kip1^ and RhoA interaction and its impact on CML have been envisioned [21]. RhoA belongs to the p21 Ras superfamily of small GTPases and like the other members shuttles between GTP and GDP bound states. RhoA is involved in a variety of signaling processes regulating cell motility [22], cytokinesis [23], smooth muscle contraction [24], and tumor progression [25,26]. Its function may thus be compared to that of a molecular switch in the cells.

We attempted to understand the importance of cytoplasmic localization of p27^kip1^ and its impact on the progression of CML from an initial chronic phase to advanced blast phase. Our results clearly indicate that cytoplasmic localization of p27^kip1^ increases with disease progression. Further, cytoplasmic p27^kip1^ interacts with RhoA and thereby regulates the activity of RhoA protein. These interactions are further guided by p210^Bcr-Abl^ and inhibition of p210^Bcr-Abl^ leads to changes in cytoplasmic localization of p27^kip1^ as well as RhoA activity. Finally, RhoA activity has a direct impact on the phosphorylation of SAPK/JNK and hence the kinase activity of the protein. In this study, we present evidence that inhibition of RhoA signaling and hence SAPK/JNK pathway promotes cell death of K562 cells in presence of imatinib.

## Materials and Methods

### Ethics statement

This study was performed with the approval of the institutional ethics committee of N. R. S. Medical College and Hospital, Kolkata 700014, India and Ramkrishna Mission Seva Pratisthan, Kolkata 700024, India as part of the project titled “CML Cell Biology- An Omics Approach”. A total of 40 patients were enrolled in this study. Written informed consents were obtained from the patients prior to bone marrow or peripheral blood sample collection.

### CML patient samples and CD34^+^ progenitor isolation

Bone marrow and peripheral blood samples were obtained from chronic and blast phase CML patients. Samples from 30 chronic phase CML patients and 16 patients in blast crisis phase of CML were obtained. Conventional cytogenetics and BCR-ABL specific quantitative real-time polymerase chain reaction (qRT–PCR) for p210^Bcr-Abl^ (b2a2) transcripts were carried out for confirmation. Characteristics of individual chronic and blast phase samples are provided in Supplementary Information S1. We considered chronic phase as having <10% blast cells and blast phase having >20% blasts in the peripheral blood or >50% blasts plus promyelocytes in bone marrow [27]. Mono nuclear cells were isolated by Ficoll-Paque (1.077, Sigma, St Louis, MO, USA), and CD34^+^ progenitors were then enriched by immunomagnetic separation (Miltenyi Biotec, Bergisch, Gladbach, Germany). Purity of CD34^+^ populations was assessed by immunostaining with CD34-PE antibody (Miltenyi Biotec) and subsequent flowcytometric analysis (FACS Calibur, Becton Dickinson, USA) [28].

### Primary cell culture and cell lines

Freshly isolated CD34^+^ cells were cultured in IMDM with 10% fetal bovine serum (FBS), 100 U/ml penicillin, 100 μg/ml streptomycin and 2 mM GlutaMAX (Invitrogen/Life Technologies, USA) and supplemented with SCF (100ng/ml), Flt3-ligand (100ng/ml) and TPO (20ng/ml). All cytokines (SCF, Flt3 ligand, TPO, GM-CSF and IL-3) were purchased from R&D systems Inc. (Minneapolis, MN, USA) were dissolved in 0.2μ filtered 1X Dulbecco PBS, pH 7.4, containing 0.1% Bovine serum albumin (BSA) and were finally used as mentioned in the text. For nulceofection of CD34^+^ cells, freshly isolated CD34^+^ primary cells were washed twice with IMDM supplemented with 2% FBS. 5x10^5^ CD34^+^ cells were nucleofected (Program; U-008, AMAXA Biosystems, USA) by taking 5 μg of respective purified plasmid DNA (Qiatip endo free maxyprep kit, Qiagen Inc, USA) for every 100 μl of cells suspended in the supplemented nucleofector solution (for detailed protocols please see: Optimized Protocol Human CD34^+^ Cell Nucleofection Kit and General Protocol for Nucleofection of Suspension Cells; Catalog No. VPA-1003 and DLA-1002, respectively, AMAXA Biosystems, USA). Before nulceofection, cells were grown overnight in IMDM supplemented with 10% FBS, human SCF (100ng/ml), Flt3-ligand (100ng/ml) and TPO (20ng/ml). After nulceofection cells were maintained in IMDM supplemented with 10% FBS, human SCF (100ng/ml), Flt3-ligand (100ng/ml), GM-CSF (10ng/ml), IL3 (10ng/ml) and TPO (20ng/ml). Efficiency of nulceofection was calculated using a GFP plasmid and was found to be >50%. Further details of the various culture conditions and materials are given in the Supplementary Information S2. Primary chronic phase CML cells were cultured in RPMI supplemented with with 10% FBS. CML cells were treated with 1µM imatinib for 24hrs. CML cell line K562 (obtained from National Centre for Cell Science, India) which constitutively express p210^Bcr-Abl^ were cultured in RPMI 1640 medium (Gibco-Invitrogen, California, USA) supplemented with 10% fetal bovine serum (Gibco-Invitrogen). Ba/F3 cells (ATCC) were cultured in RPMI 1640 medium (Gibco-Invitrogen) supplemented with 10% fetal bovine serum (Gibco-Invitrogen) and 10% WEHI culture media (as a source of IL3). Imatinib (Roche, Reinach (BL), Switzerland) was used at a concentration of 1µM for 24hr unless specified otherwise. JNK inhibitor (SP600125) was obtained from Calbiochem (Merck Millipore, Darmstadt, Germany) and used at a concentration of 20µM for 24hr. C3 exozyme was obtained from Cytoskeleton Inc. (Denver, CO, USA) and was used at a concentration of 1µg/ml.

### Real Time PCR

Total RNA was extracted from CD34^+^ cells using Tripure isolation reagent (TRIZOL, Roche, GmbH, Mannheim, Germany) and subsequently treated with RNAse free-DNAse (Roche) and quality assayed (Eppendorf BioPhotometer, Germany). The cDNA was prepared by reverse transcribing 100 ng of total RNA with reverse transcriptase reagents (Roche, GmbH, Mannheim, Germany). Quantitative PCR was subsequently performed using SYBR Green core PCR reagents (Roche) and HPRT1 was used as the endogenous control. cDNA obtained from K562 cells was used to produce the standard curve against which the ratio of gene/HPRT1 was determined. The qRT–PCR reactions and analyses were carried out in 7500 Sequence Detection System (Applied Biosystems). Details of the primer sequence are given in the Supplementary Information S2.

### Western blot analysis

For western blotting, cells were harvested and lysed in cold RIPA buffer supplemented with protease and phosphatase inhibitor cocktail (Roche, Mannheim, Germany). Equal amounts of protein was then run on parallel lanes of an SDS PAGE and blotted on PVDF membrane (GE Amersham, UK). Following incubation with primary and HRP conjugated secondary antibodies, the blots were developed using Enhanced Chemiluminescent substrate (Pierce). Antibody against p27^kip1^, phospho p27 ^kip1^S10 and RhoA were purchased from Abcam (MA, USA). Antibody against ROCK1 and ROCK2 was obtained from Santa Cruz Biotechnology Inc. (SantaCruz, USA). Primary antibodies against phospho p44/42 MAPK, p44/42 MAPK, phospho SAPK/JNK, SAPK/JNK, phospho c-jun and c-jun and cleaved caspase3 were purchased from Cell Signaling Technology (Denvers, USA). Antibody against cytochrome c was obtained from BD Biosciences (CA, USA).

### Co-immunoprecipitation

100-200μg of cell lysate was incubated with 3-4μl of anti- p27 ^kip1^/RhoA antibody at 4°C for 2hr. 20μl of Protein A/G agarose bead (Santa Cruz) was added and incubated overnight at 4°C. The immunoprecipitate was obtained by centrifugation at 5000rpm for 5min. The immunoprecipitate was washed thrice and the remaining immunoprecipitate was resolved on SDS-PAGE followed by Western blotting with anti- RhoA/p27^kip1^ antobody. GTP bound RhoA (active) was assayed using a RhoA Activation Assay Kit (Cell Biolabs, Inc., San Diego, CA, USA) according to the manufacturer’s protocol followed by western blotting with anti RhoA antibody.

### Cell fractionation and detection of membrane association of RhoA

Cells were lysed in lysis buffer containing 20 mM Tris (pH 8.0), 250mMsucrose, 0.5 mM PMSF and protease inhibitors with rapid freeze-thaw cycles. Samples were analyzed for total protein content and 200μg of each sample was then subjected to centrifugation at 100,000g for 1 h at 4ᴼC in a Beckman Ultracentrifuge. The supernatant was collected as membrane free cytosol. The insoluble pellet was washed twice with the lysis buffer and resuspended in Laemmli sample buffer and allowed to sit on ice for 1 h [29]. Samples were then processed for western blotting. For the membrane associated RhoA-nucleotide exchange experiment, cells were lysed in buffer containing 20 mM Tris (pH 8.0), 250mM sucrose, 0.5 mM PMSF and protease inhibitors with 40μM EDTA, 10 mM MgCl_2_, in the presence or absence of 100μM GTPγS and were kept at 30ᴼC for 30 min for nucleotide exchange. The lysates were then processed as above.

### Confocal imaging

Cells were washed thrice in Phosphate buffered saline (PBS). The cells were fixed for 30 min in 4% formaldehyde (Merck, NJ, USA). Cells were washed thrice with PBS and permeabilized in 0.2% TritonX100 (Sigma) with 10% FBS. After 1hr, cells were washed thrice with PBS and incubated with PE conjugated anti-cytochrome c primary antibody (BD biosciences) overnight at 4°C. After washing in PBS, cells were visualised in the confocal mode in a Carl Zeiss LSM 510 Meta microscope.

### Cell viability (Metabolic activity through MTT assay) measurement

MTT assay was performed with Cell Proliferation Kit I (MTT) (Roche, Reinach (BL), Switzerland). Briefly, cells were incubated with MTT reagent (final concentration 0.5mg/ml) for 2-3hrs. At the end of incubation period the dye was solubilized with acidic isopropanol (0.04M HCl in absolute isopropanol). The absorbance of the dye was measured at 570nm wavelength with background subtraction at 650nm.

### Statistical analysis

Statistical significance of the difference between the different conditions was assessed using a student’s two-tailed *t*-test. All mRNA expression study were analysed on independent cohorts of samples.

## Results

### Cytoplasmic localization of p27^kip1^ is dependent on p210^Bcr-Abl^ activity

**Figure 1:**
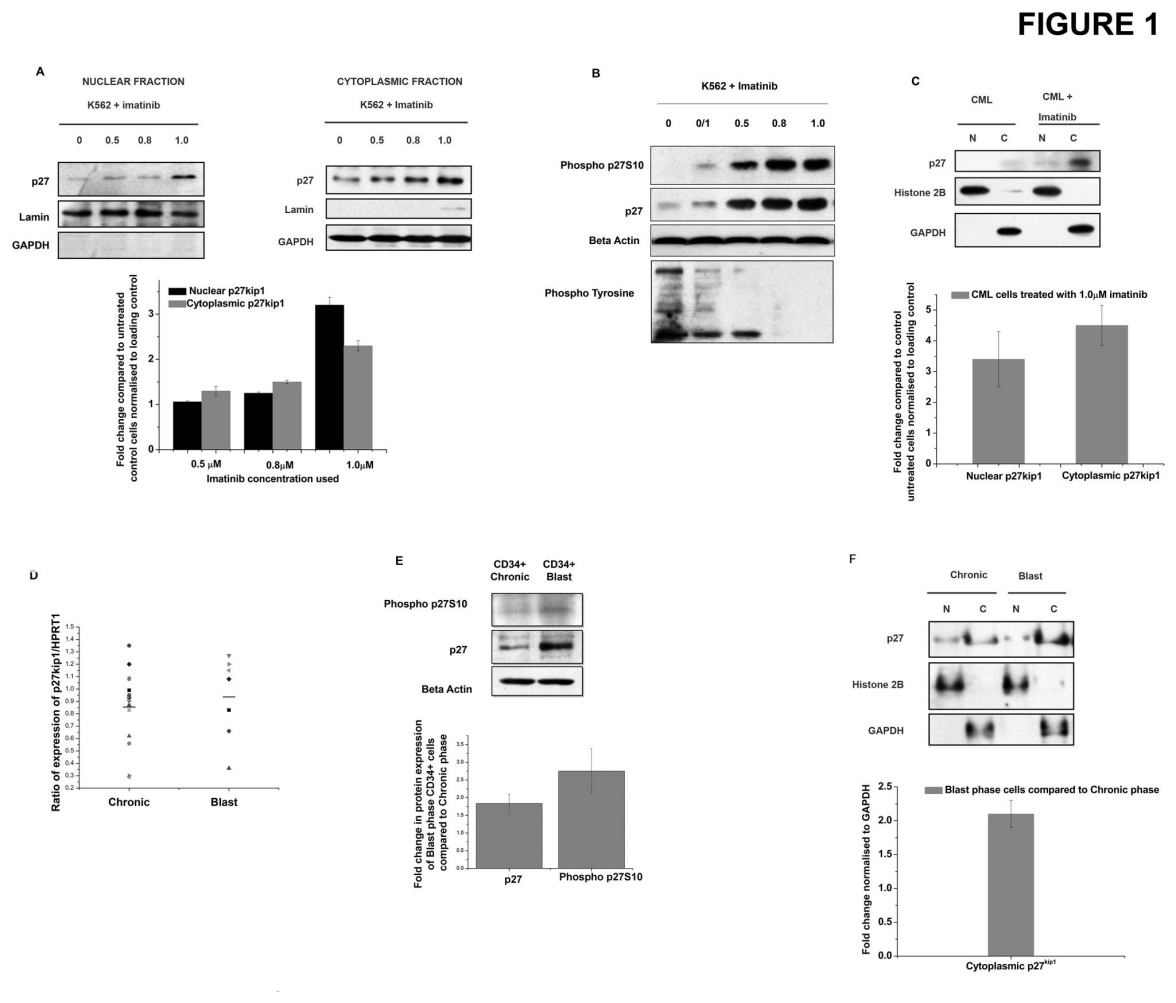
Cytoplasmic p27^kip1^ is dependent on p210^Bcr-Abl^. (A) K562 cells treated with the indicated doses (in µM) of imatinib for 24hrs were subjected to sub-cellular fractionation and the nuclear and cytoplasmic fractions were used to detect the levels of p27^kip1^. Lamin and GAPDH were used as loading controls for nuclear and cytoplasmic fractions respectively. Densitometric analysis indicates the mean ± s.e.m of three independent experiments with p<0.007 (B) K562 cells were treated with imatinib with the indicated dose (in µM) for 24 hr and the levels of phosphorylated p27 ^kip1^S10 and p27^kip1^ was assessed by western blotting. Beta actin was used as a loading control. (C) Primary chronic phase CML cells were treated with/without imatinib and then subjected to nuclear and cytoplasmic fractionation. The fractions were used to detect the levels of p27^kip1^. Histone 2B and GAPDH were used as loading controls for nuclear and cytoplasmic fractions respectively. The panel shows a densitometric analysis of three independent experiments with mean±s.e.m (p<0.01). (D) mRNA expression of p27^kip1^ in chronic and blast phase CD34^+^ stem and progenitor cells of CML was assessed by qRT-PCR. The expression was normalised against HPRT1. Horizontal bar indicates the mean of expression for the individual phase. (E) Protein expression of p27 ^kip1^S10 and p27^kip1^ in chronic and blast phase CD34^+^ cells. Beta actin was used as a loading control. Densitometric analysis of the blots shows the mean ± s.e.m of 4 individual experiments (p<0.02). (F) Primary chronic and blast phase CML cells were subjected to nuclear and cytoplasmic fractionation. The fractions were used to detect the levels of p27^kip1^. Histone 2B and GAPDH were used as loading controls for nuclear and cytoplasmic fractions respectively. The panel shows a representative blot of two independent experiments. Densitometric analysis of two independent experiments has been shown with mean±s.e.m . Lower panel shows the fold change in expression of p27^kip1^ in the cytoplasm of Blast phase CML cells compared to Chronic phase CML cells.

To check the effect of p210^Bcr-Abl^ activity on the cytoplasmic localization of p27^kip1^, we used a p27^kip1^ ser10 phosphorylation (p27 ^kip1^S10) specific antibody to determine the level of cytoplasmic p27^kip1^ since phosphorylation of p27^kip1^ at ser10 is known to induce its cytoplasmic localization [30]. We also checked the efficiency of phospho-p27 ^kip1^S10 specific antibody (Supplementary Information S1). K562 cells which express p210^Bcr-Abl^ were subjected to sub cellular fractionation and p27^kip1^ levels in nuclear and cytoplasmic fractions were determined. p27^kip1^ was found to be mostly cytoplasmic (Supplementary Information S3A). When these cells were treated with imatinib (a p210^Bcr-Abl^ tyrosine kinase inhibitor) followed by immuno-cytochemistry and confocal microscopy based imaging study, p27^kip1^ was found to localise both in the nucleus as well as in the cytoplasm of the cells (Supplementary Information S3B). To understand whether inhibition of p210^Bcr-Abl^ activity could affect the localisation of p27^kip1^, K562 cells were treated with the indicated dose of imatinib and then subjected to sub-cellular fractionation. Results revealed increased nuclear and cytoplasmic localisation of p27^kip1^ with increasing dose of imatinib (Figure 1A). This was further verified by antibody based detection of a cytoplasmic localisation specific p27 ^kip1^S10 phosphorylation (Figure 1B). We also verified the efficacy of imatinib treatment by checking for pan-tyrosine phosphorylation as well as tyrosine phosphorylation of known targets of p210^Bcr-Abl^ like STAT5 and STAT3 (Supplementary Information S3C). Additionally, treatment of primary chronic phase CML cells with imatinib was also found to increase cytoplasmic p27^kip1^ levels (Figure 1C). Thus, cytoplasmic localization of p27^kip1^ is dependent on p210^Bcr-Abl^ activity. Next, to see whether chronic to blast phase transition of CML is associated with changes in p27^kip1^ levels, the mRNA levels of p27^kip1^ was checked in the CD34^+^ stem and progenitor cells isolated from bone marrow and peripheral blood of CML patients. The data shows no significant change in the mRNA expression levels of p27^kip1^ during the disease progression (Figure 1D). To further check whether there is any change in the protein levels of p27^kip1^ and hence its cytoplasmic localization, total p27^kip1^ and phospho p27 ^kip1^S10 levels were assessed in CD34^+^ cells of CML patients. A clear increase in both total p27^kip1^ as well as phospho p27 ^kip1^S10 levels was seen (Figure 1E). This was further validated by checking the p27^kip1^ levels in the nuclear and cytoplasmic fractions of primary chronic and blast phase CML cells. Again, increased cytoplasmic accumulation of p27^kip1^ was detected in two independent sets of chronic and blast phase CML samples (Figure 1F). However, no significant change was observed in the mRNA levels of skp2 between the two phases of CML (Supplementary Information S3D). Thus, cytoplasmic localization of p27^kip1^ which is dependent on p210^Bcr-Abl^ kinase activity increases in the CD34^+^ stem and progenitor cells during chronic to blast phase transition of CML.

### Cytoplasmic p27^kip1^ interacts with RhoA

**Figure 2:**
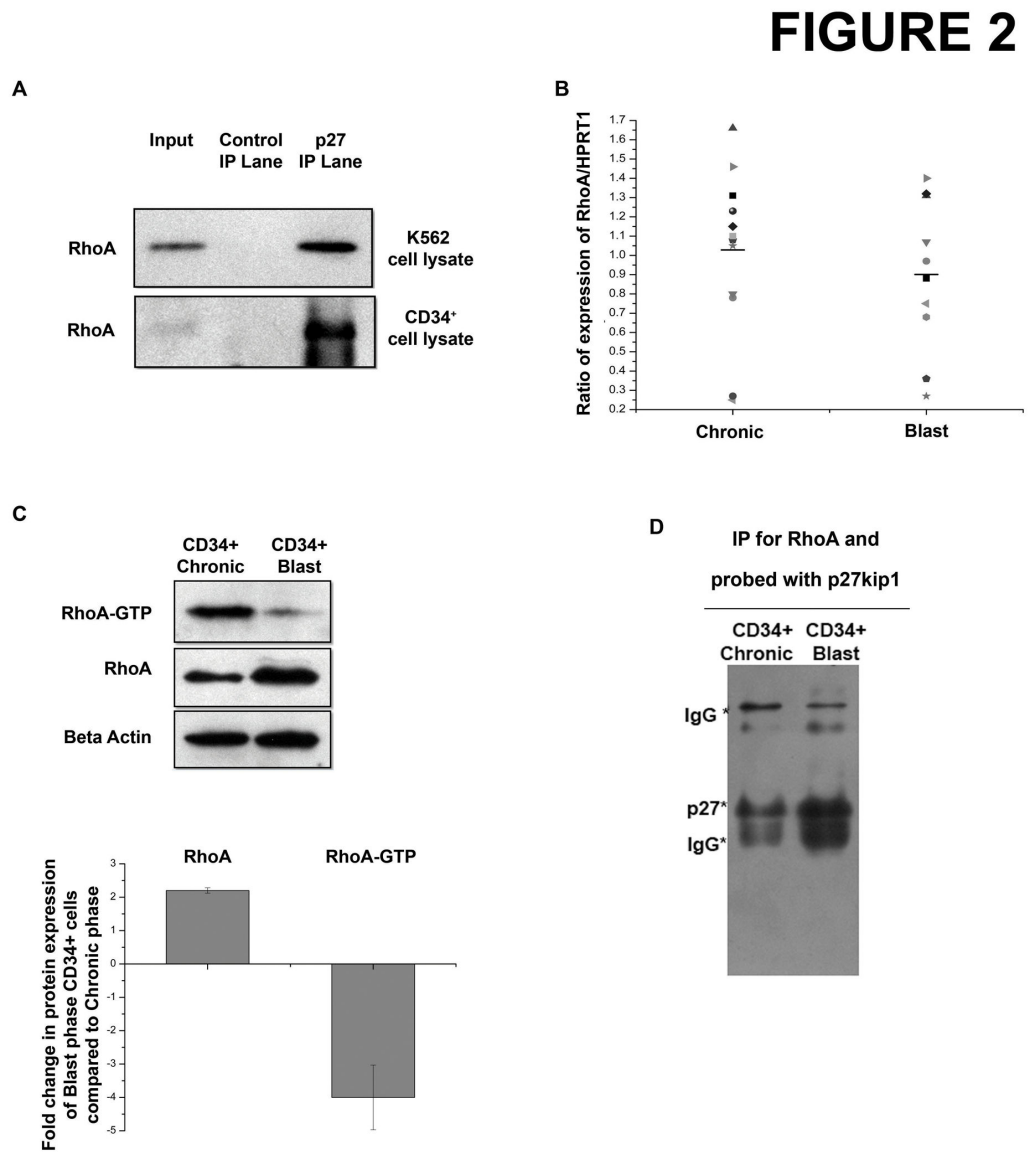
p27^kip1^ interacts with RhoA. (A) p27^kip1^ was immuno-precipitated from K562 cells and CD34^+^ cells of chronic phase CML followed by detection of RhoA in the resulting immuno-precipitate by western blotting. p27^kip1^ and RhoA interaction was confirmed in three individual Chronic phase CML samples. (B) mRNA expression of RhoA in chronic and blast phase CD34^+^ stem and progenitor cells of CML was assessed by qRT-PCR. The expression was normalised against HPRT1. Horizontal bar indicates the mean of expression for the individual phase. (C) Western blot showing the expression of RhoA-GTP and total RhoA in CD34^+^ cells of chronic and blast phases of CML. Densitometric analysis of the blots shows the mean ± SEM of 4 individual experiments for total RhoA (p<0.001) and RhoA-GTP (p<0.01). (D) Comparative immuno-precipitation assay in which equal amounts of lysates from CD34^+^ cells of Chronic and Blast phase CML was used to immuno-precipitate RhoA. p27^kip1^ was detected in the resulting immuno-precipitate. A total of three chronic and three blast phase CML samples were used to confirm the interaction.

To further understand the function of p27^kip1^ in the cytoplasmic compartment of these cells, the interaction of p27^kip1^ with a cytoplasmic protein RhoA was studied. Our results show that p27^kip1^ interacts with RhoA in K562 cells as well as in CD34^+^ cells isolated from chronic phase CML patients (Figure 2A and Supplementary Information S3E). Next, we determined whether chronic to blast phase progression of the disease is accompanied by changes in RhoA levels. qRT-PCR data shows no significant change in the mRNA expression of RhoA (Figure 2B). However, a significant increase in the protein levels of RhoA (Figure 2C) was observed. Since, the activity of RhoA is determined by its GTP bound form; we looked into the RhoA-GTP levels in the CD34^+^ cells of chronic and blast samples. Results showed a decrease in active RhoA (RhoA-GTP) levels during the progression of CML from chronic to blast phase (Figure 2C). Also, p27^kip1^ and RhoA interaction itself increased in the blast phase CD34^+^ cells compared to CD34^+^ cells from chronic phase (Figure 2D). Thus, p27^kip1^ was found to interact with RhoA in CD34^+^ cells.

### p210^Bcr-Abl^ activity affects RhoA GTP levels

**Figure 3:**
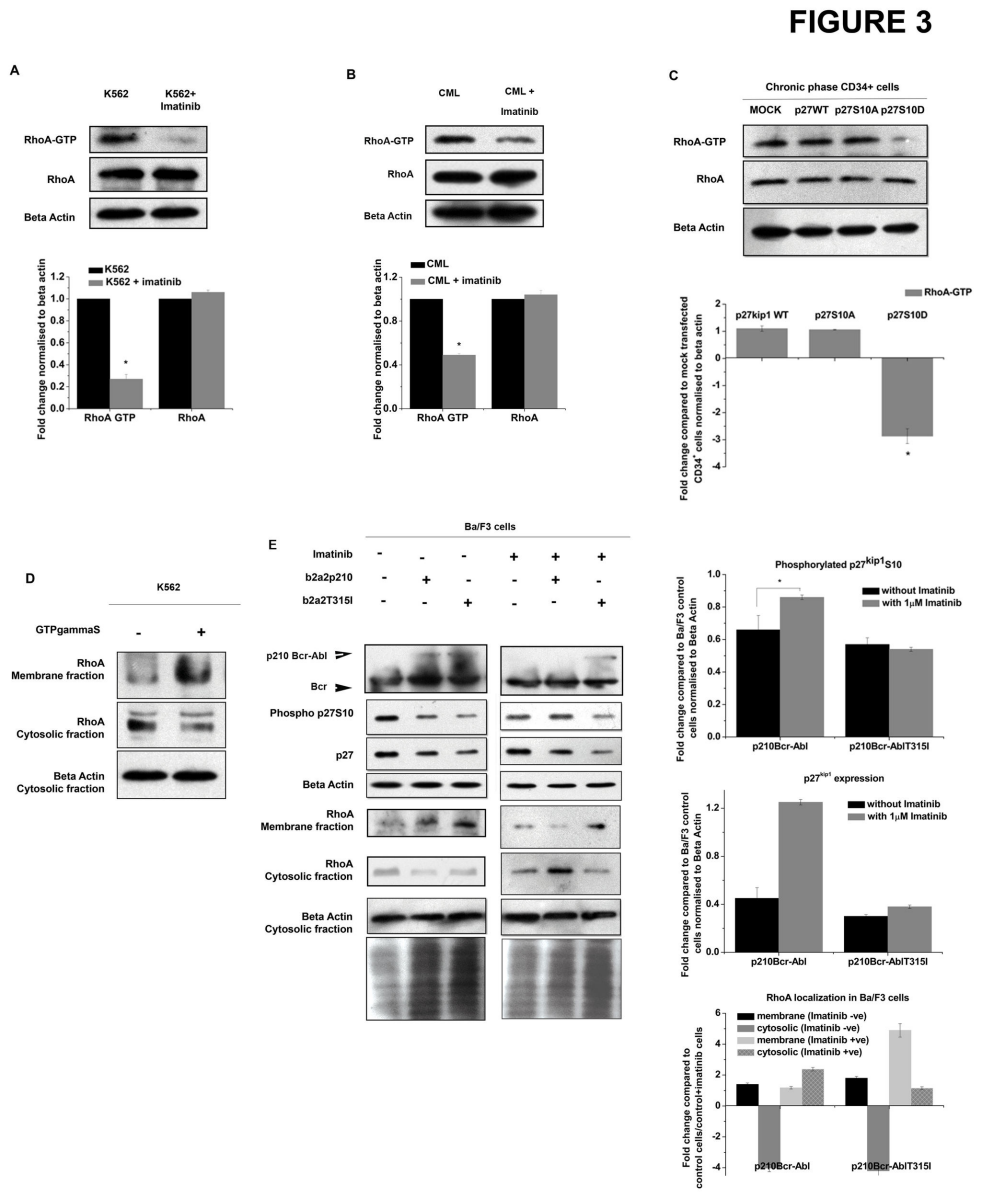
p210^Bcr-Abl^ activity affects RhoA GTP levels. (A) Western blot showing the RhoA-GTP and total RhoA levels in K562 cells treated with/without imatinib. Corresponding densitometric analysis was performed after normalisation against beta actin (n=3, *p<0.004). (B) Western blot showing the RhoA-GTP and total RhoA levels in CML cells treated with/without imatinib. Corresponding densitometric analysis was performed after normalisation against beta actin (n=3, *p<0.0014). (C) CD34^+^ stem and progenitor cells isolated from chronic phase CML bone marrow was nucleofected with the various constructs of p27^kip1^. The levels of RhoA-GTP and total RhoA were assessed in the resulting cells by western blotting. A representative blot of three independent experiments is shown (*p<0.09). (D) The distribution of RhoA between the membrane and cytosolic fraction under conditions of nucleotide exchange in K562 cells as observed by western blotting. (E) Ba/F3 cells were electroporated with p210 ^Bcr-Abl^WT and p210 ^Bcr-Abl^T315I and then treated with/ without imatinib. The western blot shows the expression of p210^Bcr-Abl^ and Bcr proteins, the distribution of RhoA between the membrane and cytosolic fraction and corresponding levels of p27^kip1^ and phosphorylated p27^kip1^ S10 in these cells. Corresponding densitometric analysis shows the mean±s.e.m of three independent experiments (*p<0.09). (All the other plots have a p<0.002).

As p210^Bcr-Abl^ activity determines the cytoplasmic levels of p27^kip1^ and since p27^kip1^ interacts with RhoA, we looked whether inhibition of p210^Bcr-Abl^ has any effect on the levels of RhoA. RhoA levels were found to be unchanged in K562 as well as CML cells when treated with imatinib. However, RhoA-GTP (active RhoA) levels decreased upon treatment with imatinib in both K562 as well as CML cells (Fig. 3A and B). Next, we looked into whether localization of p27^kip1^ had any effect on the activity of RhoA (RhoA-GTP levels). CD34^+^ cells from chronic phase CML patients were nucleofected with either mock vector, p27^kip1^ WT, p27 ^kip1^S10D (cytoplasmic localizing p27^kip1^) or p27 ^kip1^S10A (nuclear localizing p27^kip1^) vector. Results show that cytoplasmic p27^kip1^ (p27 ^kip1^S10D) reduced the RhoA-GTP levels in CD34^+^ cells obtained from chronic phase CML (Fig. 3C) indicating that cytoplasmic p27^kip1^ indeed reduces RhoA activity. RhoA is known to localize near the cell membrane and relay signals to various pathways in response to external stimulus. We thus looked into the distribution of RhoA in the cytosol and membrane fractions. Using non hydrolysable GTP analogue (GTPγS) we found that RhoA-GTP preferentially localised more in the membrane fraction (Figure 3D). In order to further investigate the effect of p210^Bcr-Abl^ on RhoA localization, Ba/F3 cells were electroporated with p210^Bcr-Abl^ WT plasmid and imatinib insensitive p210 ^Bcr-Abl^T315I plasmid and then treated with imatinib to check the membrane and cytosolic distribution of RhoA along with total p27^kip1^ and phosphorylated p27 ^kip1^S10 levels. Results show that both p210 ^Bcr-Abl^WT and p210 ^Bcr-Abl^T315I decreased the expression of total p27^kip1^ as well as phosphorylated p27 ^kip1^S10. When these cells were treated with imatinib, p210 ^Bcr-Abl^WT transfected cells showed similar p27^kip1^ and phosphorylated p27 ^kip1^S10 levels as control imatinib treated cells. p210 ^Bcr-Abl^T315I transfected cells however continued to express lower levels of both total p27^kip1^ as well as phosphorylated p27 ^kip1^S10. Therefore, cells expressing greater levels of phosphorylated p27^kip1^ and hence having increased cytosolic p27^kip1^ should have lower RhoA activity and hence lower RhoA membrane retention. Our results show that indeed, RhoA cytosolic localization increased significantly in p210^Bcr-Abl^ WT electroporated cells upon treatment with imatinib when compared to p210 ^Bcr-Abl^T315I imatinib treated cells (Figure 3E). Additionally, RhoA levels decreased in the membrane fraction of CD34^+^ cells during the progression of CML from chronic to blast phase (Supplementary Information S3F). Moreover, RhoA activity is intricately linked to filamentous actin content within a cell; increased RhoA activity leading to increased filamentous actin content. In our study, Ba/F3 cells expressing p210 ^Bcr-Abl^WT or p210 ^Bcr-Abl^T315I showed increased filamentous actin content when compared to control cells (Supplementary Information S3G). This again indicates increase in RhoA activity in cells transfected with p210^Bcr-Abl^. Together, the data clearly indicates that RhoA-GTP levels are regulated by p210^Bcr-Abl^ through its effect on cytoplasmic localization of p27^kip1^.

### RhoA activity affects cell survival

**Figure 4:**
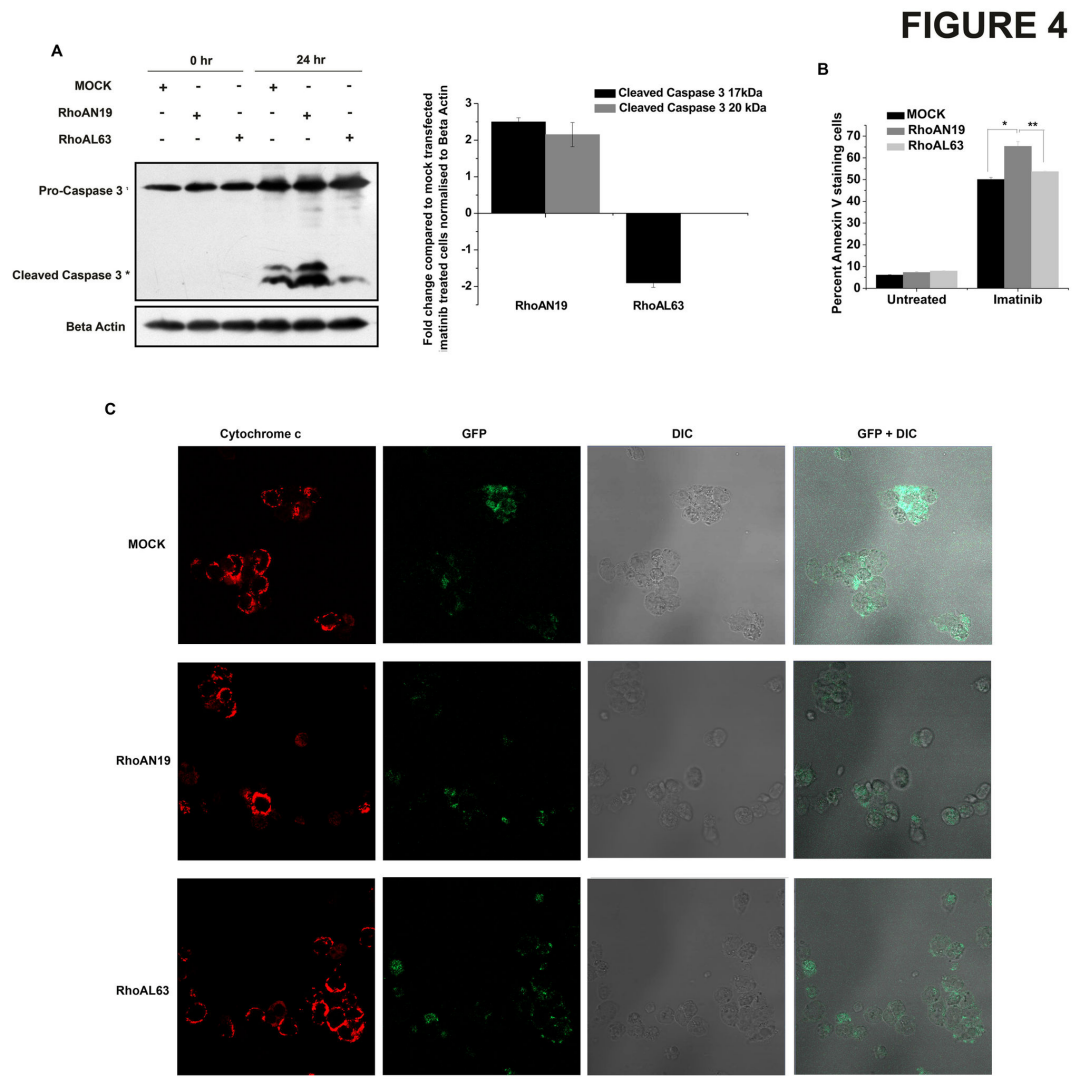
RhoA activity affects cell survival. (A) K562 cells were transfected with MOCK, RhoAN19 or RhoAL63 vectors followed by treatment with 1µM imatinib for the indicated time points in hours. Cell lysates were assessed by western blot for the presence of pro-caspase3 and cleaved active caspase3. Beta actin was used as a loading control. Corresponding densitometric analysis indicates the mean±s.e.m of three independent experiments, (p<0.02). (B) K562 cells were transfected with MOCK, RhoAN19 or RhoAL63 vectors followed by treatment with/without 1µM imatinib. Cell survival was assessed by Annexin V staining. The plot shows the percent Annexin V positive staining population in three independent experiments (*p<0.005, **p<0.008) (C) K562 cells were transfected with GFP tagged MOCK, RhoAN19 or RhoAL63 vectors followed by treatment with/without 1µM imatinib for 24 hr. Cells were stained with anti cytochrome c antibody (red) and observed under the confocal microscope. The panel shows cytochrome c staining (red), GFP (green), differential interference contrast (DIC) image and a merged image of GFP and DIC. Release of cytochrome c from mitochondria gives a diffused cytochrome c staining as against the punctate staining pattern observed in cells with intact mitochondrial membrane permeability.

The classical function of RhoA involves its interaction with proteins of the actin cytoskeleton remodelling complex. To elucidate the functional significance of the interaction between RhoA and p27^kip1^, the expression of the downstream target of RhoA - ROCK was checked. The expression of both ROCK1 and ROCK2 was found to decrease with increasing dose of imatinib (Supplementary Information S3H). Additionally, no significant difference in fibronectin adhesion was observed in CD34^+^ stem and progenitor cells expressing p27 ^kip1^S10D compared to control cells (Supplementary Information S3I). Also, CD34^+^ cells nucleofected with either p27 ^kip1^WT, p27 ^kip1^S10A or p27 ^kip1^S10D showed reduced migration in an *in vitro* transwell migration assay (Supplementary Information S3J). Therefore, it was evident that p27^kip1^ – RhoA interaction may be driving other pathways. Since active RhoA (RhoA-GTP) levels were found to decrease upon treatment with imatinib; we sought to investigate whether RhoA activity may by itself affect cell survival. K562 cells were transfected with GFP tagged mock vector, dominant negative RhoA (RhoAN19) or constitutively active RhoA (RhoAL63) and were then treated with/without imatinib. The transfection efficiency was checked under confocal microscope and was found to be ≥70%. Results show that RhoAN19 induced cells showed increased cell death when compared to control vector as observed by greater cleavage of pro-caspase3 into active caspase3 (Figure 4A). Additionally, RhoAN19 transfected K562 cells when treated with imatinib showed increased Annexin V staining when compared to mock transfected or RhoAL63 transfected cells (Figure 4B). Further RhoAN19 transfected cells showed increased cytochrome c release from the mitochondria upon treatment with imatinib as observed by diffuse cytochrome c staining indicating an increase in cell death when compared to mock transfected cells (Figure 4C and Supplementary Information S3K). This was further probed by detection of released cytochrome c in the mitochondria free cytosolic fraction of mock, RhoAN19 and RhoAL63 transfected cells +/- imatinib. Again, RhoAN19 transfected K562 cells when treated with imatinib showed greater cytochrome c release when compared to mock transfected or RhoAL63 transfected cells (Supplementary Information S3L). Thus, in the presence of imatinib, RhoA activity was found to affect cell survival.

### Inhibition of p210^Bcr-Abl^ leads to increased JNK activity

**Figure 5:**
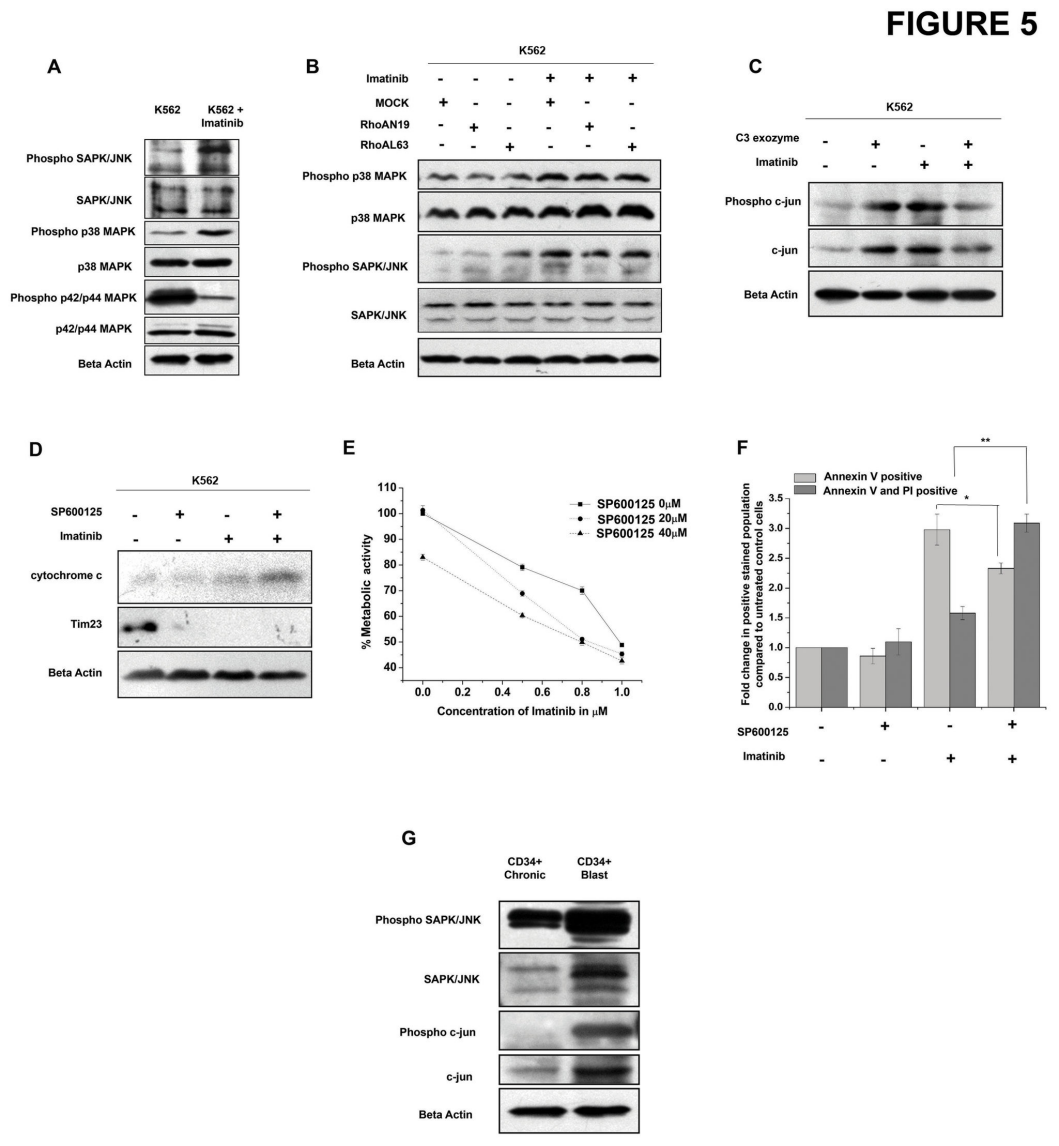
Inhibition of p210^Bcr-Abl^ leads to increased JNK activity. (A) K562 cells were treated with imatinib with 1µM for 24 hr and the levels of phosphorylated p38 MAPK, p38MAPK, phosphorylated SAPK/JNK, SAPK/JNK phosphorylated p42/44 MAPK, p42/44 MAPK was assessed. Beta actin was used as a loading control. (B) K562 cells were transfected with MOCK, RhoAN19 or RhoAL63 vectors followed by treatment with/without 1µM imatinib for 24 hr. The levels of phosphorylated p38 MAPK, p38MAPK and phosphorylated SAPK/JNK, SAPK/JNK was assessed by western blotting. (C) K562 cells were treated with imatinib, C3 exozyme or both and the activity of SAPK/JNK pathway was assessed by looking into the phosphorylation of c-jun. (D) K562 cells were treated with imatinib, SP600125 or both and the level of cytochrome c released from the mitochondria was analysed in the mitochondria free cytosolic fraction. Tim23 was used to estimate mitochondrial contamination in the mitochondria free cytosolic preparations while beta actin was used as a loading control. (E) K562 cells were treated with the indicated concentrations of imatinib and SP600125 either singly or in combination for 24hrs. The percentage metabolic activity was assessed by MTT assay. Data shows the mean percent metabolic activity for individual treatments in three separate experiments. (F) K562 cells were treated with 1µM imatinib, 20µM SP600125 or both and apoptosis was measured by staining the cells with Annexin V and PI. Graph shows the change in Annexin V stained and Annexin V + PI stained population compared to untreated control cells (n=3, *p<0.037, **p<0.002). (G) Protein expression of phosphorylated SAPK/JNK, SAPK/JNK, phosphorylated c-jun and c-jun in CD34^+^ cells of chronic and blast phase CML. Beta actin was used as a loading control.

To identify the plausible signalling pathways activated upon inhibition of p210^bcr-abl^ and the accompanying cell death, the MAPK pathway was checked. We found an increase in phosphorylation of SAPK/JNK and p38 MAPK indicating an activation of both these pathways in imatinib treated K562 cells. However, phosphorylation of p42/44 MAPK decreased with imatinib treatment (Figure 5A and corresponding densitometric analysis in Supplementary Information S3M). As RhoAN19 was found to increase cell death in presence of imatinib, we checked the association between RhoA and SAPK/JNK and p38 MAPK pathways. Results showed that RhoAL63 activates the SAPK/JNK pathway and not the p38 MAPK pathway. Also, treatment with imatinib alone was sufficient to induce SAPK/JNK phosphorylation as the phosphorylation levels were high even in RhoAN19 transfected cells treated with imatinib when compared to untreated counterpart. However, RhoAN19 transfected cells showed marginal decrease in SAPK/JNK phosphorylation when compared to mock transfected or RhoAL63 transfected cells (Figure 5B and corresponding densitometric analysis in Supplementary Information S3N and S3O). If active RhoA could indeed induce SAPK/JNK phosphorylation and hence increase SAPK/JNK signalling even in the presence of imatinib, then blocking RhoA activity should reverse these effects. Our results show that indeed exozyme C3 transferase (a specific RhoA inhibitor) in the presence of imatinib could significantly reduce SAPK/JNK signalling as observed by reduced phosphorylation of c-jun (Figure 5C and corresponding densitometric analysis in Supplementary Information S3P). Thus, SAPK/JNK activity appeared to affect cell survival. To see whether inhibition of SAPK/JNK activity could affect cell survival in presence of imatinib, K562 cells were treated with SP600125 (a specific JNK inhibitor) in the presence/absence of imatinib. Treatment with SP600125 was found to increase released cytochrome c levels as assessed in mitochondria free cytosolic fractions (Figure 5D and corresponding densitometric analysis in Supplementary Information S3Q). Also, K562 cells treated with various concentrations of imatinib and SP600125 showed greater reduction of cell viability as observed from their metabolic activity when compared to singly treated cells (Figure 5E). Further, cell death measurement by Annexin V- Propidium iodide dual staining revealed increased apoptosis in cells treated with both SP600125 and imatinib (Figure 5F). Thus, SAPK/JNK pathway was found to be involved in mediating cell survival and its inhibition increased the apoptotic cell death accompanying imatinib treatment. We next checked the levels of these proteins in the CD34^+^ cells in chronic and blast phases of CML. We found an increase in the expression of phosphorylated SAPK/JNK as well as phosphorylated c-jun indicating hyper activation of this pathway during CML progression (Figure 5G and corresponding densitometric analysis in Supplementary Information S3R). Additionally, in-vitro treatment of primary chronic phase CML cells with imatinib was also found to increase phosphorylation of c-jun (Supplementary Information S3S). Thus, SAPK/JNK activity is an important determinant of cell survival in the presence of p210^Bcr-Abl^ kinase inhibitor like imatinib.

## Discussion

p27^kip1^ has emerged as a key player in CML biology. However, its cytoplasmic localization and the consequences thus incurred remains to be elucidated. In this paper we provide evidence of its cytoplasmic localization in CD34^+^ stem and progenitor cells of CML patients. Further we show that the cytoplasmic localization of p27^kip1^ increases with the phase of the disease. Previous reports have indicated that p210^Bcr-Abl^ regulates the expression of p27^kip1^ [10–12,31]. However, the type of regulation appears to be cell specific. Whereas expression of p210^Bcr-Abl^ in cell lines was found to reduce p27^kip1^ levels [10,32,33] its expression in CD34^+^ cells lead to an increase in total cellular p27^kip1^ [13,34]. Again, inhibition of p210^Bcr-Abl^ in cell lines like K562 has been shown to dramatically increase the expression of p27^kip1^ [31] while in CD34^+^ cells imatinib has been shown to reduce the expression of total cellular p27^kip1^ [13]. Nevertheless, all the studies point towards the crucial role of p210^Bcr-Abl^ activity in the regulation of p27^kip1^. We observed a similar effect in our study; inhibition of p210^Bcr-Abl^ in primary CML cells and K562 cells increased the total p27^kip1^ as well as cytoplasmic p27^kip1^ expression. Incidentally, p27^kip1^ levels were reported to increase in both the nuclear as well as the cytoplasmic compartment of K562 cells in the presence of p210^Bcr-Abl^ inhibitor imatinib [14].

Comparison between chronic and blast phase of CML revealed no significant change in the mRNA expression of p27^kip1^ in the CD34^+^ cells. However, the protein levels showed significant increase in the expression of p27^kip1^. Previous reports have indicated that p210^Bcr-Abl^ induces increased cytoplasmic expression of p27^kip1^ in CD34^+^ cells [13]. We found that not only is the cytoplasmic localization of p27^kip1^ dependent on p210^Bcr-Abl^ activity, its level also increases with the severity of CML.

A key cytoplasmic interactor of p27^kip1^ is a member of small GTPases- RhoA. The interaction between cytoplasmic p27^kip1^ and RhoA has been widely studied in fibroblasts and has been shown to be crucial in inducing changes in the actin cytoskeleton network leading to altered cell migration [16,19,20]. Although, their interaction is well established in epithelial cells, no previous report of their interaction exists in the haematopoietic system. In this study, we report for the first time the interaction of p27^kip1^ and RhoA in CD34^+^ cells. We also show that this interaction decreases the RhoA-GTP levels. RhoA levels were found to be unchanged between the two phases of CML at the mRNA level. However, the protein expression of RhoA showed significant increase in the blast phase compared to chronic phase CML. Additionally, RhoA-GTP levels decreased with increasing phase of the disease. Together, the increase in p27^kip1^ and RhoA expression in the blast phase as well as increased interaction between the two proteins accounts for the reduction in RhoA-GTP levels. This effect was again demonstrated when cytoplasmic p27 ^kip1^S10D was nucleofected into CD34^+^ cells and a decrease in RhoA-GTP levels was observed. Additionally, the increase in both p27^kip1^ and RhoA levels along with increased interaction of p27^kip1^ and RhoA in the blast phase CD34^+^ cells compared to chronic phase cells without a simultaneous increase in RhoA-GTP levels would suggest a p27^kip1^-RhoA-GDP interaction. In fact p27^kip1^ is the only known RhoA interacting protein that has been observed to interact with both RhoA-GTP as well as RhoA-GDP [19].

p210^Bcr-Abl^ has been known to activate RhoA [35]. The DH domain of p210^Bcr-Abl^ can directly regulate RhoA activity [36]. Alternatively, p27^kip1^ is known to interact with RhoA. This interaction has been shown to decrease the activity of RhoA by interfering with its interaction with GEF [19]. Furthermore, p210^Bcr-Abl^ transfected Ba/F3 cells are also known to harbour decreased phosphorylation of RhoGDI and hence increased RhoA activation [37]. In our study, we also observed a correlation between p210^Bcr-Abl^ activity, cytoplasmic p27^kip1^ expression and RhoA-GTP levels. This correlation was evident in Ba/F3 cells which when transfected with p210^Bcr-Abl^ showed reduced cytoplasmic p27^kip1^ expression along with increased filamentous actin content. Moreover, treatment of K562 cells with imatinib also induced a decrease in RhoA-GTP. Another level of regulation of RhoA activity involves its distribution within the cells. Previous reports indicated that localization of RhoA near the plasma membrane is crucial for its activity [38–40]. We thus checked the membrane and cytosolic distribution of RhoA and found that it was not only dependent upon the activity of RhoA (RhoA-GTP) but also on p210^Bcr-Abl^. Crucial to this finding was the distribution of RhoA in the CD34^+^ cells of chronic and blast phases of CML. Membrane localized RhoA levels decreased with increasing severity of the disease which correlated with reduced RhoA-GTP levels.

The dynamics of p27^kip1^ and RhoA interaction in the presence of imatinib made us check whether RhoA may also influence cell death. Our results show that indeed, dominant negative RhoAN19 increased cell death in the presence of imatinib. To further determine the downstream molecules involved in this process, the expression of ROCK was checked. ROCK are kinases working directly downstream of RhoA [41]. Their expression has been found in the haematopoietic cells and the expression is greater in leukemic cell lines when compared to normal bone marrow or peripheral blood mononuclear cells [42]. However, since the expression of both ROCK1 and ROCK2 decreased upon inhibition of p210^Bcr-Abl^ with imatinib, we looked at other plausible pathways that may be affected by RhoA. One of the classical pathways involved in the regulation of cell survival and apoptosis is the MAPK pathway. p210^Bcr-Abl^ has been known to activate the three canonical MAPK pathways – p38 MAPK, SAPK/JNK and p42/44 MAPK. Also, transformation mediated by p210^Bcr-Abl^ has been known to require JNK and its downstream effecter c-jun [43,44]. Our study shows that although phosphorylation of p38 MAPK and SAPK/JNK increased upon treatment with imatinib, phosphorylation of p42/44 MAPK decreased suggesting a possible involvement of p38 MAPK and SAPK/JNK pathways in determining cell survival in the presence of imatinib. Additionally, SAPK/JNK pathway showed altered phosphorylation with alteration of RhoA activity. In fact, interaction between RhoA and the SAPK/JNK pathway has been reported previously in several systems [45–47]. In our study, RhoA acts as a key molecule affecting the kinase activity of SAPK/JNK. Moreover, comparison between the two phases of CML showed greater phosphorylation of SAPK/JNK in blast phase CD34^+^ cells compared to chronic phase cells. The increased expression of p210^Bcr-Abl^ in the blast crisis cells may explain the increased phosphorylation of these proteins [48]. JNK has been known to mediate survival of BCR-ABL mediated transformed B lymphoblasts [49]. Again, effect of RhoA on cell survival appears to be context dependent as some reports suggest that inhibition of RhoA increased apoptosis [50,51] while others suggest the opposite [52,53]. In our study, active RhoA (RhoA-GTP) was found to increase JNK phosphorylation and hence reduce apoptotic cell death in imatinib challenged K562 cells. Thus, inhibition of SAPK/JNK using SP600125 was found to increase apoptosis in combination with imatinib. Incidentally, the expression of RhoA has been reported to increase in imatinib resistant cells [54] and our result thus explains this observation.

The present study thus highlights the importance of increased expression of cytoplasmic p27^kip1^ during chronic to blast phase transition of CML. p210^Bcr-Abl^ dictates the dynamics of interaction of p27^kip1^ and RhoA and thus facilitates the rapid change in activity of RhoA when challenged by a tyrosine kinase inhibitor like imatinib. Under these conditions, CD34^+^ stem and progenitor cells may thus use the activated RhoA to initiate signalling through the SAPK/JNK pathway for cell survival.

## Supporting Information

Supplementary Information S1 **Characteristics of individual chronic and blast phase samples.** The age and gender of patients along with the source of samples, prior therapy, and percentage of blast cells in peripheral blood of each patient enlisted in this study has been documented in this file.
(XLS)

Supplementary Information S2 **Supplementary materials and methods.** Contains the sequences of all the qRT-PCR primers along with detailed protocols for CD34^+^ stem and progenitor cell culture, trans-well migration assay and fibronectin adhesion assay.
(DOC)

Supplementary Information S3 **Additional supplementary information.** Contains the additional supplementary qRT-PCR data, western blots and their densitometric analyses and fluorescent microscopic images.
(PDF)
