# Supplementary material for: Increased Cytoplasmic Localization of p27kip1 and Its Modulation of RhoA Activity during Progression of Chronic Myeloid Leukemia
Source: PLoS One. 2013 Oct 1;8(10):e76527. doi: 10.1371/journal.pone.0076527 (PMC3788125; doi:10.1371/journal.pone.0076527)
Supplement: Supplementary Information S2 — Supplementary materials and methods. Contains the sequences of all the qRT-PCR primers along with detailed protocols for CD34+ stem and progenitor cell culture, trans-well migration assay and fibronectin adhesion assay. (DOC) [file pone.0076527.s002.doc]

**Supplementary materials and methods S2**

List of qRT-PCR primers:

| Gene | Primer sequence |
| --- | --- |
| p27^kip1^ | 5’TGATCCGTCGGACAGCCAGA 3’  5’CCGTCTGAAACATTTTCTTCTGT 3’ |
| skp2 | 5’AGAGGAGCCCGACAGTGAGA 3’  5’GGGTGGCCCAGGTTTGAG 3’ |
| DYRK1b | 5’ATCTTGCTGTGCAACCCCAA 3’  5’CGGTAGAAGCGGCTCTGGAAT 3’ |
| KIS1 | 5’TGAGAAACGAGAGCACGAGAAA 3’  5’TTTGTGCCTCTCGGTTCTCTT 3’ |
| RhoA | 5’CATCCGGAAGAAACTGGT 3’  5’TCCCACAAAGCCAACTC 3’ |
| ROCK1 | 5’GAATGACATGCAAGCGCAA 3’  5’GAGGTCCAAAAGTTTAGCAGC 3’ |
| ROCK2 | 5’TCCATAAAGCTCTCTCGGCG 3’  5’GCTGGTGAAACCTCTCTGTCA 3’ |
| HPRT1 | 5’GACACTGGCAAAACAATGCAGAC3’  5’ TGGCTTATATCCAACACTTCGTGG3’ |

Culture of CD34^+^ cells: All primary cells were cultured in IMDM (Iscove’s Modified Dulbecco Medium, Stem Cell Technologies, Canada) supplemented with 10% fetal bovine serum (FBS), 100 U/ml penicillin, 100 μg/ml streptomycin and 2 mM GlutaMAX (Invitrogen/Life Technologies, USA). All cytokines (SCF, Flt3 ligand, TPO, GM-CSF and IL-3) were purchased from R&D systems Inc. (Minneapolis, MN, USA) were dissolved in 0.2μ filtered 1X Dulbecco PBS, pH 7.4, containing 0.1% Bovine serum albumin (BSA) and were finally used as mentioned in the text. Freshly isolated CD34^+^ primary cells were washed twice with IMDM supplemented with 2% FCS and 5x10^5^ cells were nucleofected (Program; U-008, AMAXA Biosystems, USA) by taking 5 μg of respective purified plasmid DNA (Qiatip endo free maxyprep kit, Qiagen Inc, USA) for every 100 μl of cells suspended in the supplemented nucleofector solution (for detailed protocols please see: Optimized Protocol Human CD34^+^ Cell Nucleofection Kit and General Protocol for Nucleofection of Suspension Cells; Catalog No. VPA-1003 and DLA-1002, respectively, AMAXA Biosystems, USA).

Fibronectin adhesion assay: Freshly isolated CD34^+^ cells were cultured overnight in the presence of SCF (100ng/ml), Flt3(100ng/ml) and TPO(20ng/ml) supplemented with 10% FBS. Cells were then nuclofected with 5µg plasmid DNA and kept in media containing GM-CSF(10ng/ml), IL3(10ng/ml), SCF (100ng/ml), Flt3(100ng/ml) and TPO(20ng/ml) for 24 hr. Equal number of cells (5x10^5^ cells/ml) were then seeded in triplicate into fibronectin (Sigma, St. Louis, MO, USA) coated wells of a chamber slide and kept for 5 hr. Subsequently, the media was aspirated out and the wells were washed with phosphate buffered saline thrice. The adhered cells were counted.

Transwell migration assay: Freshly isolated CD34^+^ cells were cultured overnight in the presence of SCF (100ng/ml), Flt3(100ng/ml) and TPO(20ng/ml) supplemented with 10% FBS. Cells were then nuclofected with 5µg plasmid DNA and kept in media containing GM-CSF(10ng/ml), IL3(10ng/ml), SCF (100ng/ml), Flt3(100ng/ml) and TPO(20ng/ml) for 24 hr. Cells were then kept in serum free media with the cytokines for 12 hr. Equal numbers of cells (5x10^5^ cells/ml) were then seeded in triplicate into transwell migration assay chambers with 3 µm pore size membrane (Millipore). The cells were seeded into the upper chamber without FBS and migration was promoted by addition of 10%FBS to the lower chamber. The numbers of cells in the upper and lower chambers were counted at the end of 16 hr on a FACS Calibur platform after staining the cells with PE conjugated anti CD34 antibody and addition of equal numbers of APC stained beads.
