# Supplementary material for: Increased Cytoplasmic Localization of p27kip1 and Its Modulation of RhoA Activity during Progression of Chronic Myeloid Leukemia
Source: PLoS One. 2013 Oct 1;8(10):e76527. doi: 10.1371/journal.pone.0076527 (PMC3788125; doi:10.1371/journal.pone.0076527)

SUPPLEMENTARY INFORMATION S3

(A) Specificity of phospho-p27^{kip1}S10 antibody as demonstrated from the western blot. Two cell lines were chosen for this assay – K562 having majorly cytosolic p27^{kip1} and AGS having majorly nuclear p27^{kip1}. Cells were subjected to sub-cellular fractionation and total p27^{kip1} levels and phospho-p27^{kip1}S10 was probed using respective antibodies in each fraction. Purity of the fractions was assessed by nuclear Lamin and cytoplasmic GAPDH levels in both the fractions.

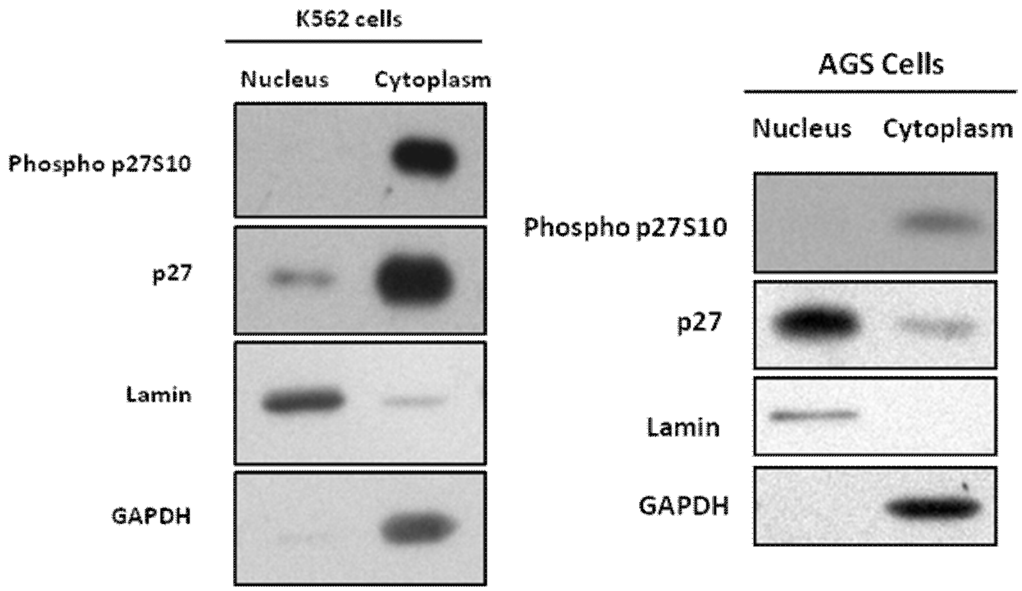

(B) Immuno-cytochemistry based imaging of p27^{kip1} in K562 cells treated with/without imatinib. Cells were stained with p27^{kip1} primary antibody followed by Alexa flour tagged secondary antibody and visualised under confocal mode. Panel shows p27^{kip1} satining, differential interference contrast (DIC) image of cells as well as a DIC and p27^{kip1} merged image.

K562

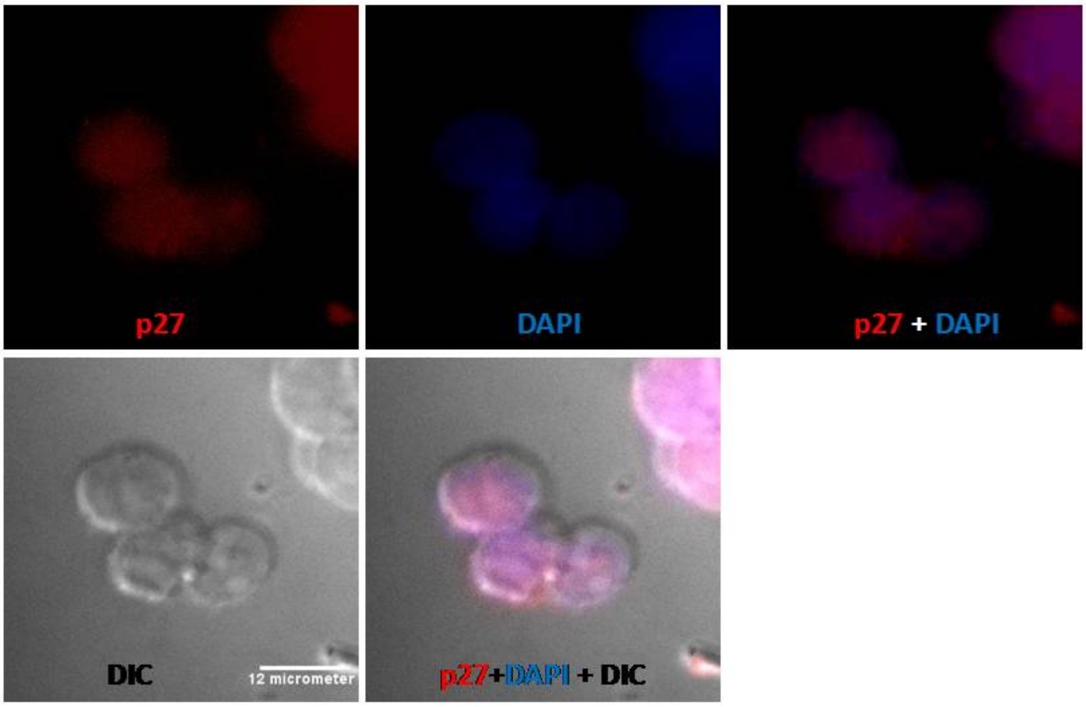

K562 + Imatinib

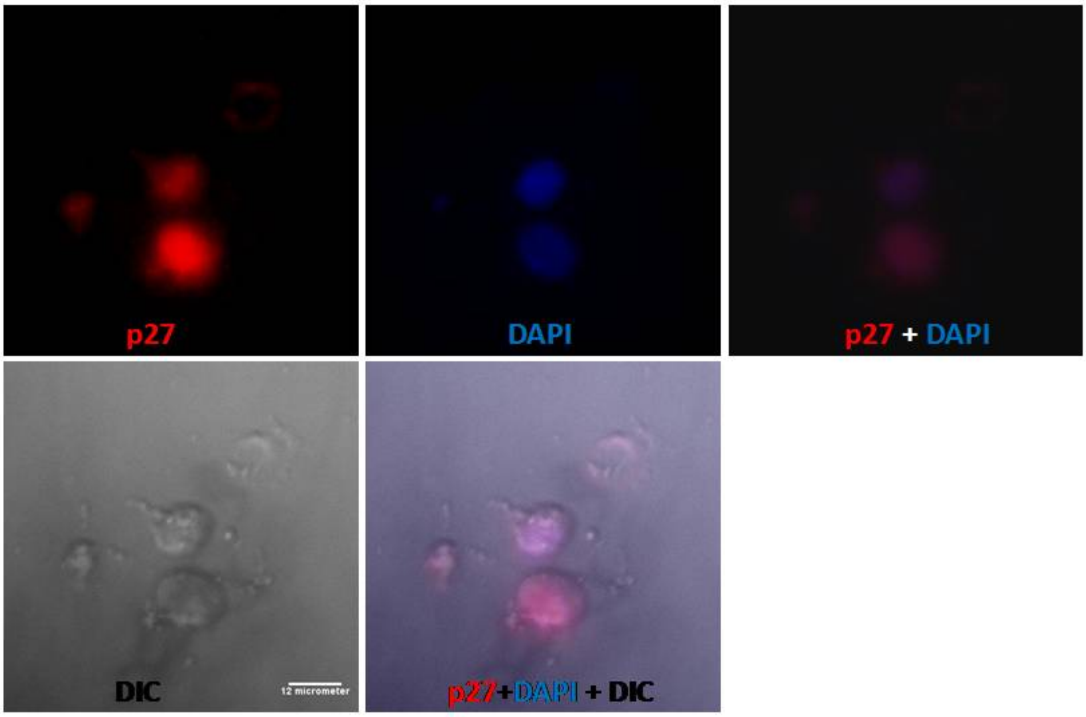

(C) Efficacy of imatinib treatment as demonstrated by phosphorylation of known targets (STA3, STAT5) and pan-tyrosine phsophorylation. Pan-threonine phosphorylation was used as negative control. Ponceau S staining of the membrane blot has been used to indicate loading.

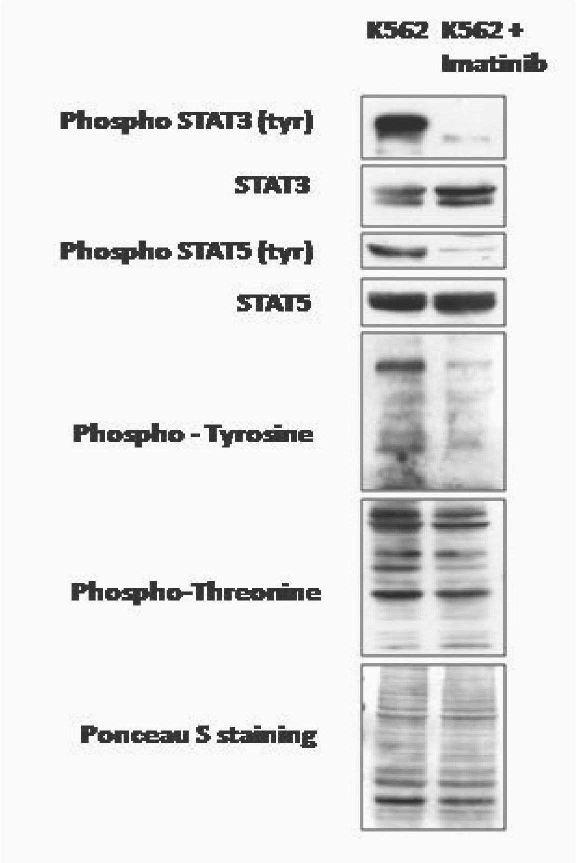

(D) mRNA expression of Skp2 normalised against HPRT1 in the $CD34^+$ stem and progenitor cells of chronic and blast phases of CML.

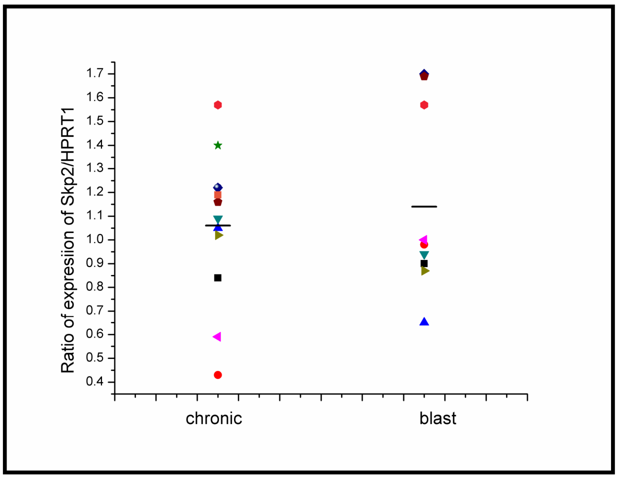

(E) RhoA was immuno-precipitated from K562 cells followed by detection of $p27^{kip1}$ in the resulting immuno-precipitate by western blotting.

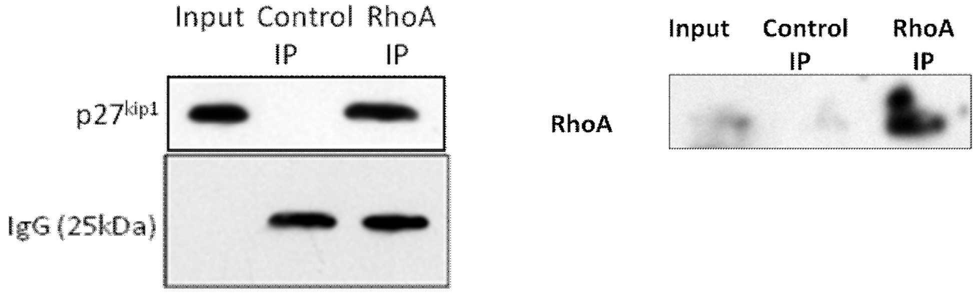

(F) Distribution of RhoA in the membrane and cytosolic fractions of $CD34^{+}$ stem and progenitor cells of chronic and blast phase CML.

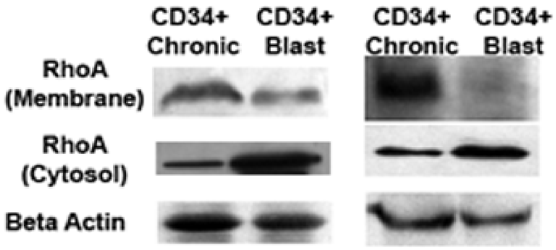

(G) Ba/F3 cells were electroporated with $p210^{Bcr\text{-}Abl}$WT and $p210^{Bcr\text{-}Abl}$T315I. The cells were fixed, permeabilized and stained with rhodamine tagged phalloidin. Expression of either of the $p210^{Bcr\text{-}Abl}$ constructs caused an increase in filamentous actin content within the cells as observed by flow cytometric analysis. The decreased phosphorylated LIMK1/LIMK2 in the presence of $p210^{Bcr\text{-}Abl}$ or $p210^{Bcr\text{-}Abl}$T315I was also observed by western blotting. Beta actin was used as a loading control. However, no discernable change in cell shape or size was evident.

i) Ba/F3 cell size and shape remains unaltered upon transfection with $p210^{Bcr\text{-}Abl}$ or $p210^{Bcr\text{-}Abl}$T315I

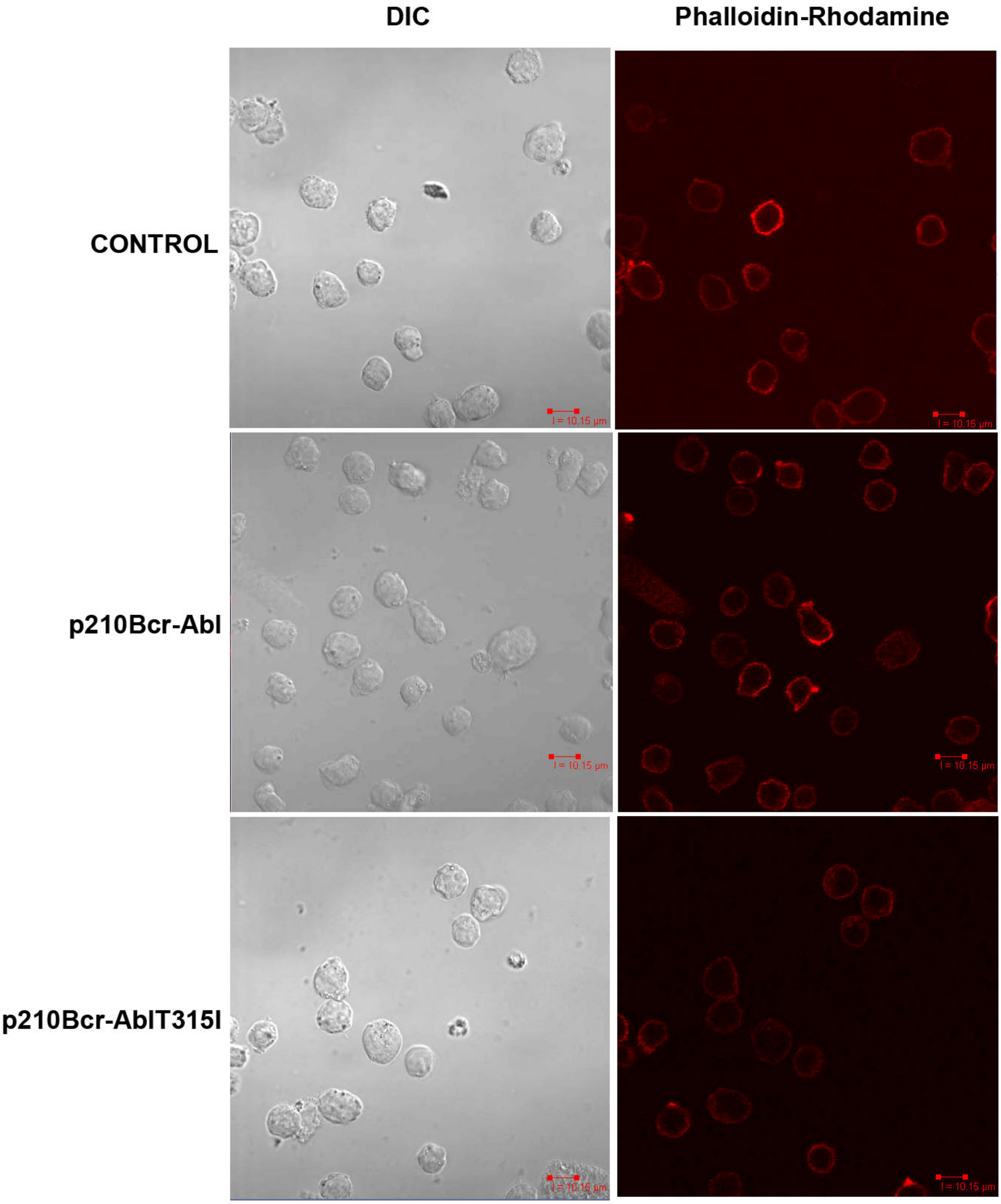
DIC
Phalloidin-Rhodamine
CONTROL
p210Bcr-Abl
p210Bcr-AblT315I
l = 10.15 µm

ii) BaF3 cells transfected with p210$^{Bcr-Abl}$ or p210$^{Bcr-Abl}$T315I show increase in filamentous actin content as observed by increased staining with rhodamine-phalloidin.

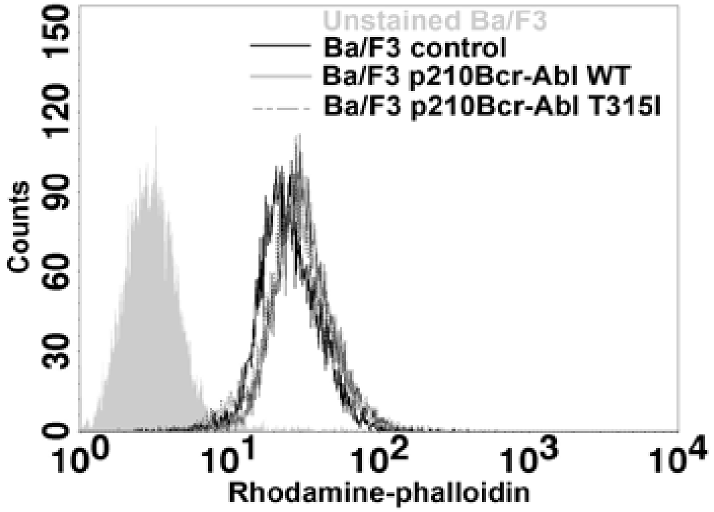

iii) Ba/F3 cells transfected with p210$^{Bcr-Abl}$ or p210$^{Bcr-Abl}$T315I show decreased expression of phosphorylated LIMK1/LIMK2. Densitometric analysis of the blot has been represented below.

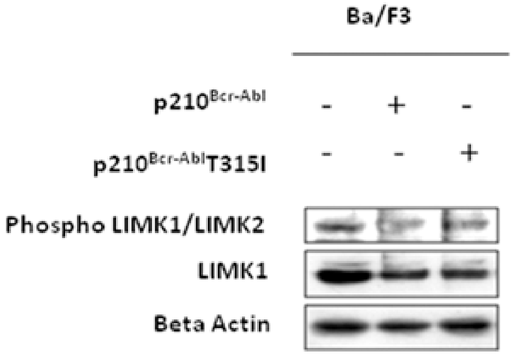

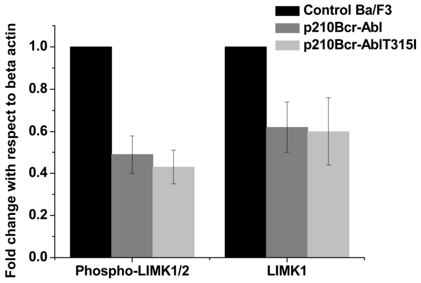

(H) K562 cells were treated with imatinib with the indicated dose (in µM) for 24 hr and the levels of ROCK1 and ROCK2 was assessed by western blotting. Beta actin was used as a loading control.

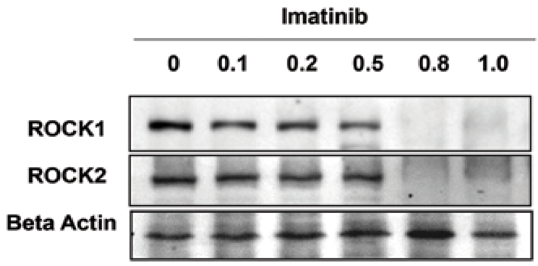

(I) Fibronectin adhesion assay: CD34[+] cells isolated from chronic CML patient was nucleofected with the various constructs of p27 and their adherence to fibronectin matrix was assayed in triplicate. Data represents fold change in cellular adhesion to fibronectin normalised against control CD34[+] cells.

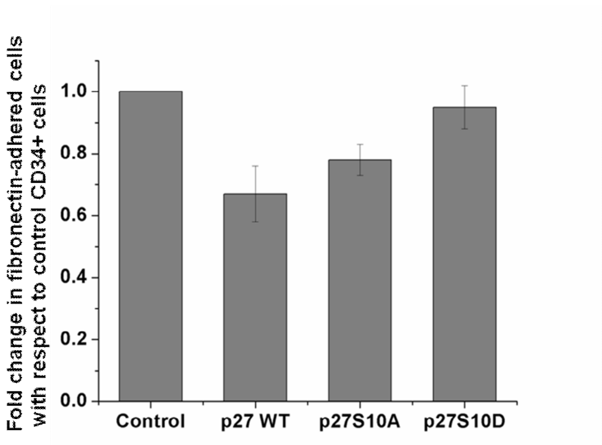

(J) Transwell migration assay: CD34[+] cells isolated from chronic CML patient was nucleofected with the various constructs of p27 and their migration through the 3µ pore was assayed in triplicate. Data represents percentage of transfected cells which have migrated to the lower chamber with respect to control CD34[+] cells.

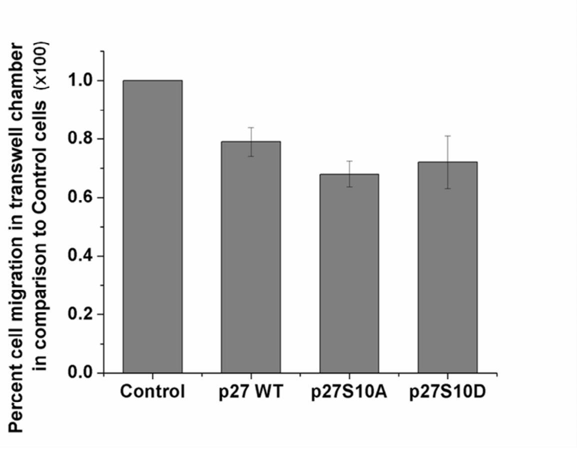

(K) K562 cells were transfected with GFP tagged MOCK, RhoAN19 or RhoAL63 vectors followed by treatment with/without 1μM imatinib for 24 hr. Cells were stained with anti cytochrome c antibody (red) and observed under the confocal microscope for diffused cytochrome c staining indicating release of cytochrome c from the mitochondria. 100 cells were observed in three independent preparations and the mean ± SEM was plotted. ($p<0.005$)

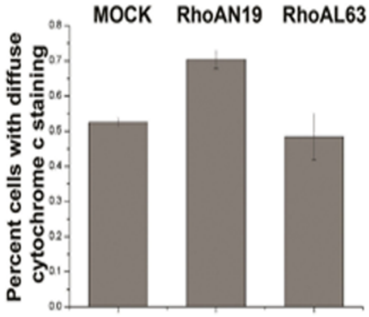

(L) K562 cells were transfected with MOCK, RhoAN19 or RhoAL63 vectors followed by treatment with/without 1μM imatinib. Cytochrome c released from the mitochondria was estimated in the purified mitochondria free cytosolic fraction by western blot. Tim23 was used to estimate mitochondrial contamination in the cytosolic preparations (densitometric estimation given in parenthesis) while beta actin was used as a loading control. ($n=3$, $p<0.019$)

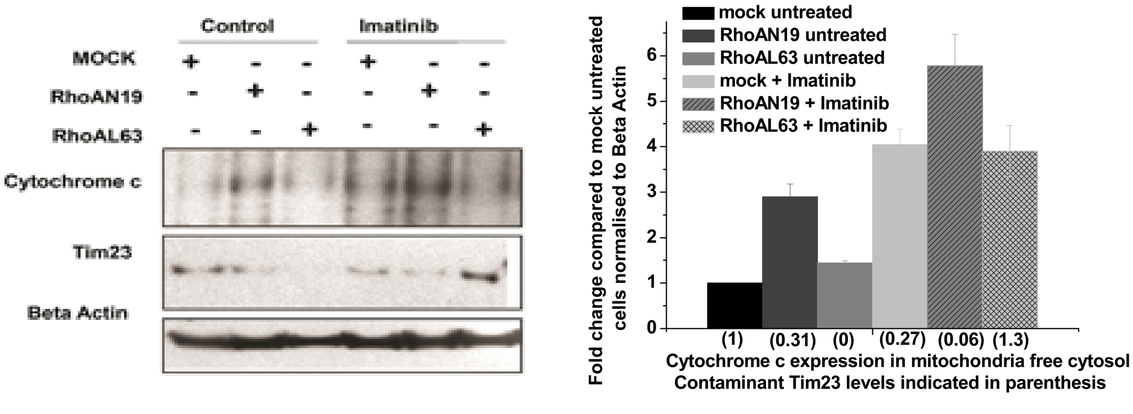

(M) Densitometric analysis of the western blots (Fig. 5A) showing the change in expression of phosphorylated p38MAPK, p42/44MAPK, SAPK/JNK and p38MAPK in the presence of imatinib in K562 cells when compared to untreated control cells. Densitometric expression values were normalised against Beta Actin expression. (n=3, *p<0.01, **p<0.004, ***p<0.006)

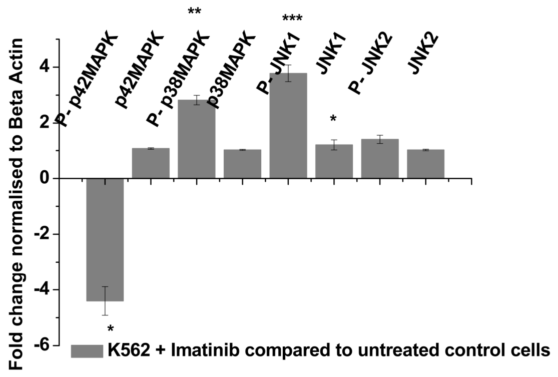

(N) Densitometric analysis of the western blots (Fig. 5B) showing the change in expression of phosphor-p38MAPK and p38MAPK with change in RhoA activity in the presence of imatinib. Normalised densitometric expression values were plotted against the expression seen in Mock transfected untreated K562 cells. (n=3, *p<0.0001, **p<0.0002, ***p<0.0003, ****p<0.003)

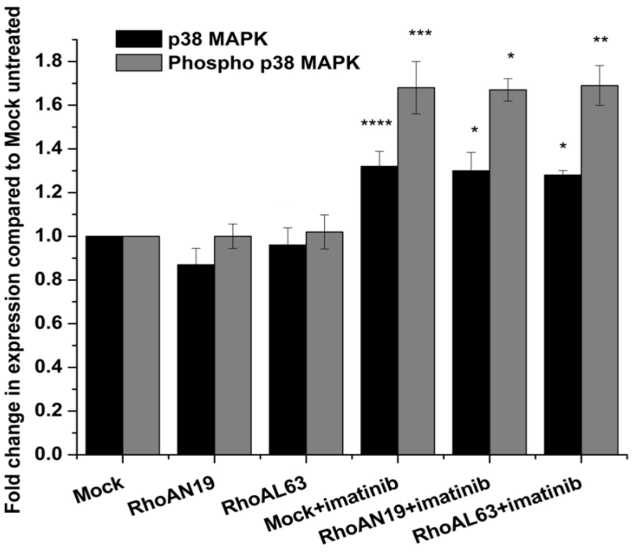

(O) Densitometric analysis of the western blots (Fig. 5B) showing the change in expression of phosphor-SAPK/JNK and SAPK/JNK with change in RhoA activity in the presence of imatinib. Normalised densitometric expression values were plotted against the expression seen in Mock transfected untreated K562 cells. (n=3, *p<0.0005, **p<0.001, ***p<0.005 and ****p<0.008)

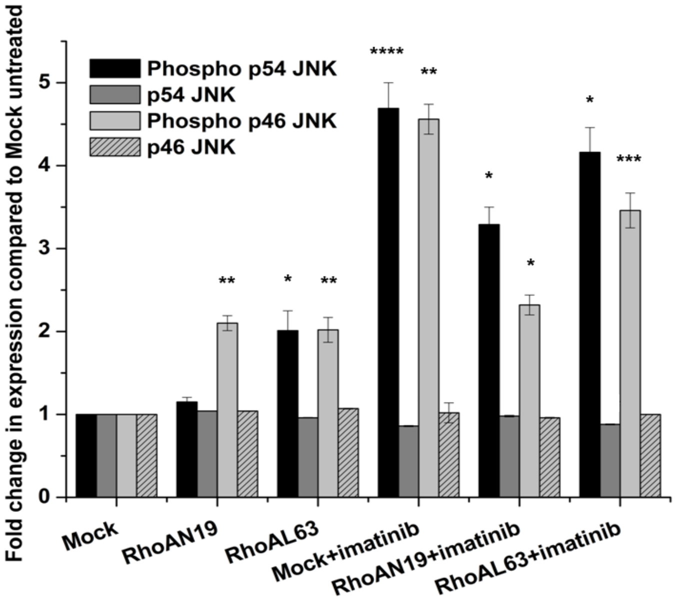

(P) Densitometric analysis of the western blots (Fig. 5C) showing the change in expression of phospho-c-jun and c-jun in the presence of imatinib or C3 exozyme or both. Normalised densitometric expression values were plotted against the expression seen in untreated K562 cells. (n=3, *p<0.016, **p<0.003)

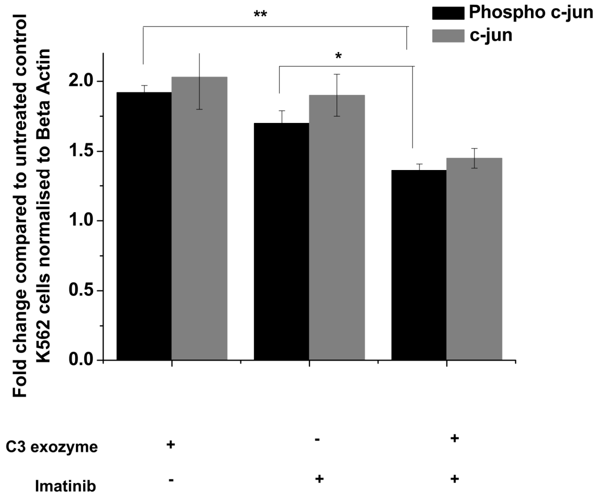

(Q) K562 cells were treated with SP600125, imatinib or both. Cytochrome c released from the mitochondria was estimated in the purified mitochondria free cytosolic fraction by western blot (Fig. 5D). Tim23 was used to estimate mitochondrial contamination in the cytosolic preparations while beta actin was used as a loading control. (n=3,p<0.008)

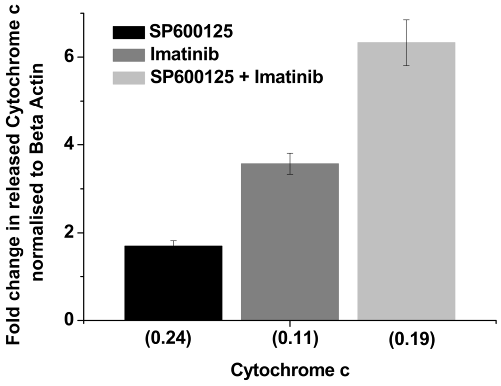

(R) CD34+ cells from Blast phase CML patients were compared to chronic phase CML patients for the expression of phosphorylated SAPK/JNK and c-jun. Densitometric analysis of the blots (Fig. 5G) shows the mean±s.e.m of three independent sample sets. ($p<0.05$). The densitometric values of the proteins or their phosphorylated forms in chronic phase were considered to be 1 and the corresponding fold change in blast phase was then calculated.

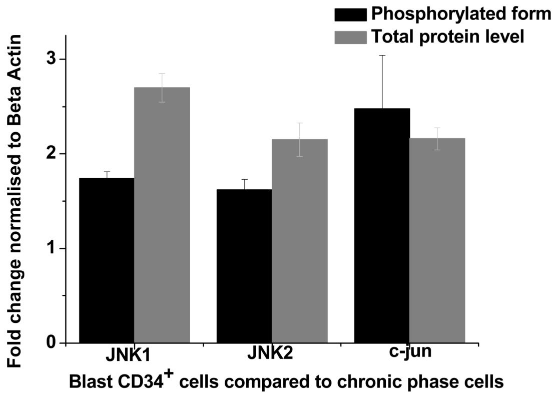

(S) The activity of SAPK/JNK pathway in primary chronic phase CML cells from two individual CML patients with/without imatinib treatment was checked. Results show increased phosphorylation of c-jun in the imatinib treated samples compared to control cells.

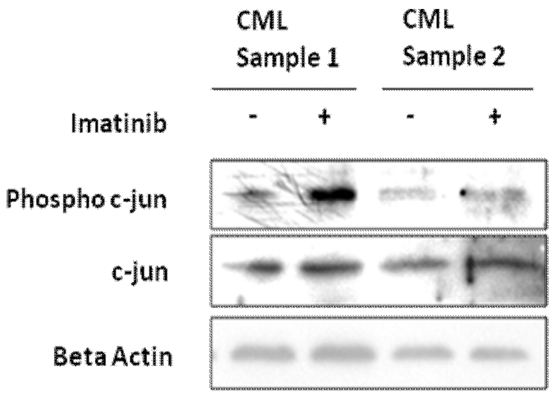

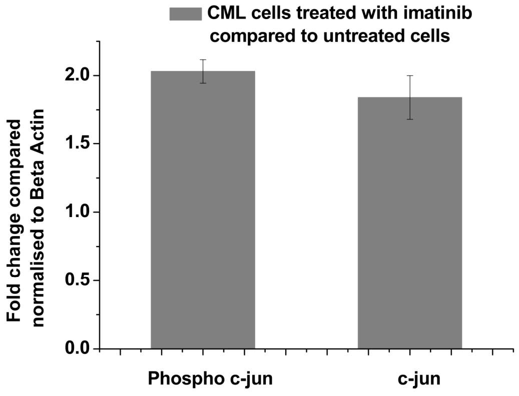

Supplement: Supplementary Information S3 — Additional supplementary information. Contains the additional supplementary qRT-PCR data, western blots and their densitometric analyses and fluorescent microscopic images. (PDF) [file pone.0076527.s003.pdf]
